# Case of Fracture of the Spinous Process of the Third Dorsal Vertebra

**Published:** 1839-02-02

**Authors:** W. Harris


*Case of Fracture of the Spinous Process of the Third Dorsal Vertebra. Reported by* W. Harris, M. D.

At my late residence in Chester county, on the 12th of May, 1833, whilst mounting a young and vicious horse, the end of an umbrella, which I held folded in my right hand, was suddenly inflated by a puff of wind, which terrified the animal, and caused him to run, plunge, and throw me violently over his head. I was thrown a somerset in the fall, and received, thereby, the whole force of the concussion upon my back between the shoulders.

A partial paralysis was immediately experienced in the diaphragm and intercostal muscles, and respiration was consequently performed with great difficulty. The greatest efforts enabled me to inflate my lungs, in a very imperfect degree only. My first impulse was to crawl to the fence and seize a post, in the hope that, by fixing my chest, I might be enabled to fill my lungs; but this effort was also vain.

As I fell within a short distance of my own mansion, and as my lower extremities were uninjured, I immediately, and without much assistance, reached my chamber, and was put to bed. My extremities, soon after, became quite cold, my pulse scarcely perceptible, my face livid, my breathing more difficult, and my feelings induced me to think that the period of my dissolution was at hand. Mustard plasters were promptly applied to my feet and wrists, my whole body was enveloped in warm flannel, and surrounded with bottles of hot water.

As my pain, especially in the region of the diaphragm, was very intense, and the feeling of suffocation distressing beyond description, in the hope of obtaining some relief, I requested my friend, Dr. Matlack, after I had taken a large draught of hot tea, to open a vein in my arm. As this operation was performed before reaction had taken place, the blood flowed very sluggishly, and only three or four ounces could be obtained. This was not followed by the slightest mitigation of my suffering. All the ordinary means for bringing about a reaction were now put in operation, and about six hours after the injury, my extremities began to grow warm, and my pulse to rise. At this time, my brother, Dr. Stephen Harris, arrived, who took from my arm about twenty ounces of blood, which was succeeded by a more easy respiration, and a slight alleviation of all my sufferings. New symptoms now made their appearance. A violent action commenced in my heart. Its pulsations were so strong that they could be felt all over the left side of the thorax, and be seen across my chamber. Gas was generating rapidly in my stomach and intestines, and my abdomen was becoming, consequently, quite tympanitic. The muscles upon the posterior and superior part of the thorax, as well as upon the posterior part of my arms and fore-arms, were paralysed. Those on the fore part of my arms were not affected. I could not stretch out my arms from my body; but if this were done by an assistant, I could flex them with considerable force. My kidneys, too, suffered from the shock, as no urine appeared to be secreted. Bed-time of the first day having now arrived, I took a large dose of cathartic medicine, which produced no sensible effect. I passed a sleepless night. The next morning, my brother, Dr. Thomas Harris, arrived from Philadelphia, who, upon examination of my back, discovered that the spinous process of the third dorsal vertebra was fractured. Motion and crepitus were plainly evident. He was apprehensive, too, as partial paralysis had followed the concussion, that effusion had taken place within the spinal sheath, and that thickening of that membrane and its attendant consequences, were to be apprehended. Accordingly, the treatment was now directed mainly to the seat of the injury. Cups were immediately applied, a row on either side of the spinous processes of the dorsal vertebræ, and about eight ounces of blood extracted. After this, attention was directed to my bowels, and, in consequence of their present torpor and distension with flatus, it was only after repeated doses of aperient medicines, at short intervals, and the administration of enemata, that an opening was effected. By this I was relieved of one of my most unpleasant symptoms—distension from flatulence. In the course of the second day, I voided, too, for the first time, a small quantity of urine.

The third day after the injury, my symptoms were all more favourable, except the palpitation of the heart. This continued with uninterrupted violence until the end of the second week. There was a peculiar tenderness or sensibility in all the muscles that covered the thorax. Every movement gave me such intense pain, that when I was turned from my back to either side, or the reverse, I had to be gently rolled over by pulling the sheet upon which I lay, and at the same time making a rotatory movement.

The same plan of treatment was continued, and my sufferings gradually subsided, so that upon the fourth week, after my spine had been cupped seven times, I began to sit up and move gently about my chamber. There was, however, great weakness in the spinal column immediately above and below the injury, and a disposition to curve forwards. I could not remain long, however, in an erect position, without a return of severe pain, which could only be relieved by my resuming the horizontal attitude. I had a jacket made with two pieces of whalebone behind, on either side of two rows of eyelet-holes, at the place of junction, which, after it was put on and tightly laced, gave me great support and comfort. I still, however, suffered more or less from the erect position, unless the weak part was supported by the back of a chair.

When I resumed my professional duties, I suffered, at times, intense pain in the injured part, and was often obliged to lie down at the houses of my patients. Bending over a patient to dress a wound or a fracture, could not be endured more than five minutes at a time. Indeed, I have repeatedly been obliged to stop once, twice, and sometimes thrice, in the midst of such dressing, and lie down upon my back for a few moments—the pain being too intense to be endured. And often, whilst bending over the table, in writing a letter, I would be seized with such intense pain that I have frequently been obliged to prostrate myself upon my back on the carpet, to procure ease. The recumbent position, it is worthy of remark, always afforded me relief. I was never afterwards able to ride on horseback, nor in any kind of carriage, comfortably, unless it had a back sufficiently high to reach the weak part. The palpitation of the heart continued at intervals. Ascending a flight of stairs, or a hill; any undue exercise of body, as well as excitement of mind, would induce it. This affection of the heart was pronounced by my friend Dr. Jackson, after a careful examination, to be hypertrophy of that organ, and by his advice, I avoided, as much as practicable, every thing of an irritating or exciting character. At irregular intervals, moreover, I had violent attacks of neuralgia, in the nerves which originated at or near the injured part, which are always accompanied with a deranged state of the digestive organs. These attacks have always been relieved in two or three days, by the following treatment: First, I take two blue pills, each containing three grains, and after a few hours, half an ounce of Epsom salts, which operate freely; then have cups applied along the spine, which I have scarified, or use them dry, accordingly as the pain is more or less intense, and as my strength will or will not admit of the loss of blood. Subsequently to this, I take Dover's powder, or some narcotic, in sufficient quantities to subdue all pain. In the commencement of an attack, the operation of a cathartic alone has occasionally given me complete relief. Again, the application of cups has removed all pain before the operation was completed. But, in most instances, purging, cupping, and narcotics, were all necessary to procure relief. Again, I have the same disease in a more mild and chronic form, which is best overcome by counter irritation, Croton oil, &c., rubbed along the spine, over the nervous roots.

In the ensuing winter, finding that I was likely to be a permanent invalid, and that I was no longer able to attend to an extensive country practice, I determined to remove to Philadelphia, and subject myself to the embarrassing alternative of re-commencing my professional career.

Since that period, I have suffered at times from the affection of my heart, and from neuralgia, but, upon the whole, my general health has gradually improved, and both these affections are less troublesome. I have ceased to wear the laced jacket, the curvature forwards is certainly less, and the weakness at the injured part nearly removed.

Last summer, after suffering six weeks from chronic neuralgia in the neighbourhood of the old injury, without obtaining any relief, I determined to try sea-bathing; and accordingly, in the month of July, I proceeded to Cape May for that purpose. During my sojourn in that place, I bathed once or twice every day, and engaged in many active exercises, especially in rolling nine-pins. I took an excursion, moreover, across the bay, to and from the Delaware break-water, by which I was made horribly sea-sick. The bathing, or the exercise, or the sea-sickness, or all combined, completely removed my neuralgia in less than two weeks, and I have suffered very little from it since.

I had two young ladies under my care, affected with the same disease, both of whom returned wonderfully relieved.

In November last, I undertook to improve my anatomical knowledge by a course of dissection. In the afternoon of the first day, I stood in a bent position over the dissecting table, for three hours, and during the last hour, felt a good deal of pain in the back. After returning home, I had the painful part ironed with a hot flat-iron, as I frequently do in slight attacks, and went to bed. After an hour I was perfectly relieved, and continued easy all night. I was now in a quandary as to the propriety of returning to the dissecting room, but concluded to renew my labour there once more. My sufferings on this occasion were much less, and, after ten days' daily labour of this kind, I could stand in a bent position over the table three or four hours at a time, without any inconvenience. The sufferings, in this instance, I believe, arose entirely from weakness in the muscles of the back. The long continued pressure upon them from the laced jacket, and the constant care of the injured part, had, in a great measure, destroyed their strength;* and continued exercise restored them to their wonted vigour. I have never felt any weakness in that part since.

It is worthy of remark, that I lost, by this injury, three-fourths of an inch of my height, which was probably owing to the absorption of some of the intervertebral cartilages, and explains the disposition, after the injury, to anterior curvature. It is probable, now, that nature has thrown out a bony splint in front of the vertebra, that has relieved this tendency.

The history of this case is narrated, with the hope that it may give comfort to some valetudinarian who may be suffering from a similar injury, as, without controversy, "misery loves company."

* This may afford a profitable hint to some of the young ladies who are destroying the strength of their muscles by pressure.

